# “What I Do Not Eat”: Feeding Difficulties in Middle Childhood—An Italian Pilot Study

**DOI:** 10.3390/nu18010129

**Published:** 2025-12-31

**Authors:** Paolo Brambilla, Laura Antolini, Marco Giussani, Carlo Agostoni, Paolo Becherucci, Emanuela Malorgio, Cristiana Berti

**Affiliations:** 1 National Board of the Società Italiana delle Cure Primarie Pediatriche, 20122 Milan, Italy; paolo.brambilla3@gmail.com (P.B.); dottormarcogiussani@gmail.com (M.G.); paolobecherucci@gmail.com (P.B.); malorgioemanuela4@gmail.com (E.M.); 2 Dipartimento di Medicina e Chirurgia, Università Milano Bicocca, 20900 Monza, Italy; laura.antolini@unimib.it; 3 IRCCS Fondazione Don Carlo Gnocchi ONLUS, 20148 Milan, Italy; 4 Centro Rischio Cardiovascolare Pediatrico Istituto Auxologico IRCCS, 20145 Milan, Italy; 5 Pediatric Unit, Fondazione IRCCS Ca’ Granda Ospedale Maggiore Policlinico, 20122 Milan, Italy; cristiana.berti2@gmail.com; 6 Department of Clinical Sciences and Community Health, University of Milan, 20122 Milan, Italy

**Keywords:** early life feeding practices, feeding difficulties, middle childhood, anthropometry, blood pressure, parents’ eating, family pediatrician

## Abstract

Background/Objectives: Feeding difficulties in childhood can persist over time, affecting health and family dynamics. Timely identification is crucial to prevent atypical eating behaviors and nutrition-related consequences. However, data on childhood feeding difficulties remain limited. This study provides the first pilot characterization of eating behaviors among Italian children aged 5–11 years in order to characterize feeding difficulties, identify protective or detrimental factors, and assess relationships with anthropometric indices or blood pressure. Methods: In 2023, a 1-year cross-sectional pilot study was launched by the Società Italiana delle Cure Primarie Pediatriche involving child–parent dyads. Family pediatricians collected anthropometric data and blood pressure, after which parents completed an online questionnaire purposely developed for this study. The questionnaire generated a feeding difficulty score (0–10) where higher values reflected greater risk of atypical eating behaviors. Scores were categorized as follows: low risk (0–1), intermediate risk (2–6), and high risk (7–10). Results: A total of 742 questionnaires were collected. Overall, 19.8% of the children were categorized as high risk (7–10) for atypical behavior, 43.8% as intermediate risk (2–6), and 36.4% as low risk (0–1). Children with two omnivorous parents showed significantly lower odds of feeding difficulty scores at or above any threshold (OR 0.46, 95% CI 0.30–0.71; *p* < 0.0001). Borderline associations were observed for a breastfeeding duration of at least 9 months (OR 0.79, 95% CI 0.61–1.02; *p* = 0.075) and baby-led weaning (OR 0.72, 95% CI 0.52–1.00; *p* = 0.053). High-risk children had a significantly lower BMI percentile with respect to the other groups. Conclusions: A significant proportion of Italian children aged 5–11 years exhibited moderate to severe risk of atypical behaviors. Parental eating appears to play a key role in shaping children’s eating behaviors in middle childhood, underscoring the pivotal role of pediatricians in guiding families. Further research and targeted strategies are needed to prevent childhood feeding difficulties.

## 1. Introduction

Childhood is the period during which eating patterns are established. Atypical feeding behaviors in children are of concern, as they occur in 20–50% of typically developing children and may become persistent, with implications for health and family dynamics [1]. In light of the rise in food-related silent pandemics among pediatric populations, i.e., malnutrition in all its forms [2] and eating disorders [3], setting up nutritionally balanced and varied dietary patterns in middle childhood is crucial. Despite inconsistencies in defining, measuring, and prevalence (ranging from 5.6 to 59%), fussy/picky eating is acknowledged as the most common feeding difficulty throughout childhood [4]. It is characterized by an unwillingness to eat familiar foods or to try new foods, along with strong food preferences, leading to a demand for specific meal preparation and/or consumption of a limited variety of foods [4]. Timely identification of feeding difficulties is critical to prevent the onset of lifelong atypical eating behaviors and nutrition-related consequences. In this context, family pediatricians can play a unique role [5]. In Italy, there is a shortage of data about general feeding difficulties among children in middle childhood, i.e., from 5 to 11 years old, which is an age at which eating habits are fairly established and any remaining difficulties can be considered to be persistent. Indeed, existing data refer to neophobia [6,7,8]. To depict the eating behavior in this population, the Società Italiana delle Cure Primarie Pediatriche (SICuPP) launched the 1-year cross-sectional project entitled “What I don’t eat”. This pilot study was planned as an explorative survey that was meant to gather preliminary information on feeding difficulties among Italian school-aged children, thus contributing to the current knowledge on the topic. It aimed to achieve the following: (1) characterize the feeding difficulties; (2) identify potential influencing factors, i.e., protective or detrimental factors; and (3) investigate the relationship between feeding difficulties and anthropometric indices or blood pressure.

## 2. Materials and Methods

### 2.1. Study Population and Study Design

This study had a cross-sectional design. It was carried out between October 2023 and October 2024 on primary school children and their parents. The inclusion criteria were as follows: children aged 5–11 years, without any significant chronic diseases requiring a specific diet (except for food allergy or intolerance or celiac disease); parents’ understanding of the Italian language, familiarity with digital tools, and written informed consent. In September 2023, the SICuPP invited the family pediatricians that were distributed in the national territory to join the study. Each pediatrician who was interested in participating received 40 single-use access codes for the Limesurvey.net platform, managed by Strategia&Controllo Srl (appointed and supervised by SICuPP), for filling in the online questionnaire. The codes could only be used once without traceability to guarantee anonymity. During routine check-ups, the pediatrician proposed the study to the parents of eligible children. After obtaining signed informed consent, parents were given an access code and a link to the dedicated platform to complete the online questionnaire independently and anonymously within 7 days. To ensure complete anonymity, Strategia&Controllo generated and anonymized the access codes in batches. The principal investigator distributed these codes to the pediatricians, who gave one code to each family. The pediatricians did not keep any records linking the codes to the respondents, which guaranteed irreversible anonymization. Neither the pediatricians nor the principal investigator had access to individual questionnaire responses.

### 2.2. Measurements

Anthropometric measurements and blood pressure were collected by the pediatrician during the check-up visit. Each child’s body weight and height were assessed using standard procedures [9]. Body mass index (BMI) was calculated as weight (kg)/height (m^2^) and transformed into percentiles. The child’s nutritional status was classified based on the International Obesity Task Force (IOTF) BMI cut-offs, adjusted for age and sex: underweight being below the 10th percentile; overweight being above the 85th percentile; and obesity being above the 95th percentile [10]. Blood pressure (BP) values were obtained with certified aneroid sphygmomanometers or automatic electronic instruments, according to the standard [11]. BP was measured at the start, middle, and end of the visit. The average of the values of the last two measurements was used. Systolic pressure (SBP) and diastolic pressure (DBP) were transformed into percentiles and the corresponding pressure category according to the American Academy of Pediatrics [11].

### 2.3. Survey

A three-section questionnaire was specifically developed to be completed by parents regarding the following:

General information and early feeding practices, including duration of breastfeeding (BF) and complementary feeding methods, i.e., traditional spoon-fed weaning or baby-led weaning (i.e., auto-weaning; BLW).

Anthropometry and BP: Parents entered values for weight, height, SBP, and DBP as measured by the pediatrician during the check-up visit.

Eating behavior: Parents reported their child’s feeding behavior by replying to 15 purposely designed questions related to difficulties, refusal, selectivity, and monotony. The first ten questions (scoring questions) were adapted from the “Food Fussiness” and “Slowness in Eating” subscales of the Child Eating Behavior Questionnaire (CEBQ) [12] to better fit the aim of our research. The CEBQ was chosen because it is a validated and widely used instrument for evaluating eating behaviors in children. The remaining five questions examined aspects that were not included in the CEBQ. These aspects included acceptance of school meals, caregiver influence on meal adherence, marketing’s effect on purchases, constant difficulty chewing, and the need for device distraction. Parents could choose between the response options ‘Yes’, ‘No’ or ‘Do not reply’. Because these domains are conceptually different, we did not consider it appropriate to combine all 15 items into a single score. Therefore, only the scoring questions were taken into account to calculate the score, as they represent a coherent construct based on a validated framework. A value of 1 was attributed to answers that were suggestive of a feeding difficulty, i.e., “No” to the first eating behavior items and “Yes” to the others. The individual scores were summed up into an overall feeding difficulty score (varying from 0 to 10), whereby the higher the score, the greater the risk of atypical feeding behavior. The score was divided into ranges as follows: a score of 0–1 indicated a low risk (LR), a score between 2 and 6 defined an intermediate risk (IR), and a score of 7–10 suggested a high risk (HR). Parents could express their food preferences among the following options: omnivorous, vegetarian, vegan, or restrictions for religious reasons.

### 2.4. Data Analysis

Descriptive statistics were used to summarize participants’ general characteristics, early feeding practices, anthropometry and BP, and eating behaviors. Continuous variables were reported as means with standard deviations, while categorical variables were expressed as frequencies and percentages. Comparison of distributions across groups was performed by the chi-square test for categorical variables and by the Wilcoxon ranks sum test for continuous variables. Associations between the risk of atypical behavior and possible explanatory factors were assessed using univariate and multiple ordinal logistic regression models, including factors that were significant at the univariate analysis in the latter model. This approach has the dual advantage of not requiring categorization of the outcome variable and eliminating the need to validate a dose–response effect when the regressors are binary. Regressors were included in the multivariate model if the results were significant in the univariate analysis. A graph was also used to illustrate possible mediators and exposure that could affect the score of eating difficulties. Statistical significance was set if the two sided *p*-value was lower than or equal to 0.05. Internal scale reliability for the ten scoring questions in the “Eating Behavior” section of the questionnaire was assessed using Cronbach’s coefficient alpha (α) [13]. Analyses were performed using Stata software (version 17 for Windows).

### 2.5. Ethical Considerations

Before starting the data collection, parents were informed about the objective of the study and the intention to publish the results. Those who were willing to participate signed a privacy policy and consent form according to the European Commission General Data Protection Regulation (679/2016). Participation in the survey was fully voluntary and anonymous, and subjects could withdraw at any time. The study was performed according to the Declaration of Helsinki [14]. This research was not considered to include either medical experimentation or direct intervention on human subjects. No invasive interventions were performed, and there was no risk to participants. An additional ethical committee review of the study protocol was considered to be unnecessary.

## 3. Results

Out of 68 family pediatricians giving their availability to participate in the study, 42 gathered data. A total of 797 surveys were collected. We included 742 participants in the final analysis after excluding 55 surveys due to missing anthropometric measures.

### 3.1. Participants’ Characteristics

Table 1 shows the socio-demographic characteristics, feeding history, and health status of participants. The population was balanced by gender, with 53.6% female and 46.4% male participants, and had a mean age (±standard deviation, SD) of 7.5 (±1.6) years. Most children were born at term (90.7%) and with an adequate weight of 3.2 (±0.6) kg. Furthermore, 457 participants (61.6%) lived in Northern Italy, 126 (17.0%) in Central Italy, and 159 (21.4%) in Southern Italy. Most children (75.1%) had siblings. One third (31.5%) were breastfed for less than three months, 11.9% were breastfed up to six months, and 30.2% were breastfed beyond the age of 12 months. Additionally, 80.6% were fed complementarily by using the traditional spoon-fed weaning. More than three fifths (65.2%) of the children were adequately nourished, 12.3% were underweight, and 22.5% suffered from over nutrition. Eighty-seven percent had normal BP, and 11.6% had hypertension. A significant relationship was found between nutritional status and blood pressure (BP) (*p* = 0.0001), with the highest prevalence of hypertension observed among overweight (23%) and obese (28%) children. Furthermore, the relationship between the hypertension status and all the parameters considered in Tables was non-significant. On average, mothers were 34.5 (±4.9) years old, and fathers 37.5 (±5.7) years old. Almost half of the mothers and one fourth of the fathers had graduated. The vast majority of parents were Italian and omnivorous.

### 3.2. Children’s Feeding Difficulty Score and Atypical Behavior Risk

Table 2 reports parents’ answers to the 15 specifically designed questions related to difficulties, refusal, selectivity, and monotony. The percentage of parent-reported answers suggestive of child feeding difficulties ranged from 24.5% to 37.2%, except for the eating behavior “4. Does your child absolutely refuse certain foods?” being 61.7%. Moreover, 24.5% of parents sometimes bought food for their child for reasons other than simple food choices, 19.2% of children did not eat everything at school, and 16.7% of children behaved differently depending on which family members offered food. The results showed a high degree of clustering among difficulties, refusal, selectivity, and monotony. Regarding the feeding difficulty score derived from the scoring questions, 147 (19.8%) children were at HR of atypical behavior, 325 (43.8%) at IR, and 270 (36.4%) at LR. Based on Cronbach’s α score of 0.79, the ten scoring questions exhibited high internal consistency [13].

### 3.3. Factors Associated with the Feeding Difficulty Score

Table 3 reports the mean value of the feeding difficulty score by the sample socio-demographic characteristics and feeding history. We observed higher scores among children who were an only child, had at least one non-omnivorous parent, were breastfed for <9 months (no differences were found when 6 and 12 months were considered as the cut-off), and were traditionally weaned.

Analysis of children’s feeding history, i.e., breastfeeding duration (<9 months versus ≥9 months) and complementary feeding method (traditional baby weaning versus BLW), showed a higher prevalence of positive answers to the eating behavior question “3. Does your child always eat the same foods?” among parents of children who were breastfed <9 months (37.9% versus 27.1%, *p* = 0.006). Similarly, difficulties with vegetables (“5. Does your child have a hard time eating vegetables or refuse?”) were more frequent in children who were breastfed <9 months (42.8% versus 30.2%, *p* = 0.002), and in those who were traditionally weaned (39.7% versus 26.4%, *p* = 0.005).

Table 4 shows frequencies and differences in children’s general characteristics and feeding history by the type of atypical behavior risk, i.e., HR versus IR versus LR. LR children had a higher probability of being children of two omnivorous parents (*p* = 0.001). In contrast, HR children were more likely to have at least one non-omnivorous parent (mother: *p* = 0.004; father: *p* = 0.009). Furthermore, HR children also tended to have been breastfed <9 months (*p* = 0.055), while LR participants tended to have no siblings (*p* = 0.068), and to have been auto-weaned (*p* = 0.061).

Table 5 reports the findings from an ordinal logistic analysis. We found an association between the odds of a score indicating eating difficulties being greater than or equal to a fixed threshold and having two omnivorous parents [0.46 (0.30–0.71); *p* < 0.0001]. A borderline impact was observed for BF duration ≥9 months [0.79 (0.61–1.02); *p* = 0.075] and BLW [0.72 (0.52–1.00); *p*-value = 0.053]. Absence of any impact was observed for only children [1.15 (0.82–1.60); *p*-value = 0.423] and first-born children [1.20 (0.89–1.61); *p*-value = 0.237].

**Figure 1:**
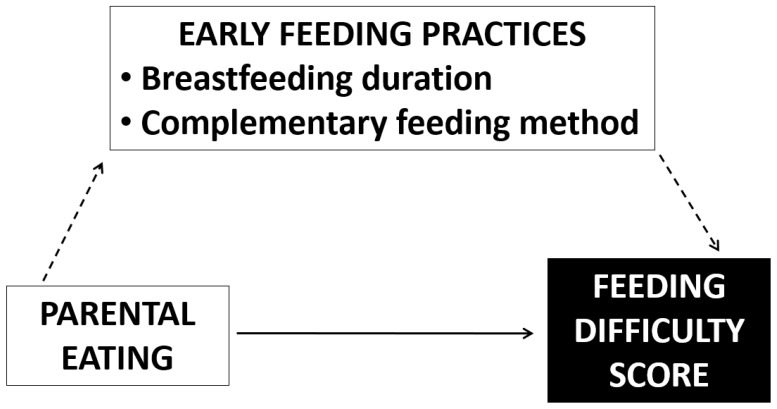
Possible mediators and exposure that could affect the score of eating difficulty.

Figure 1 illustrates early feeding practices (BF duration and type of weaning) as potential mediators of parental eating. This formulation aligns with the observed, though not statistically significant, impact of parental eating on BF duration and type of weaning, where having two omnivorous parents appeared to be associated with an increased likelihood of BF for ≥9 months (44.6% vs. 40.5%; *p*-value = 0.504) and a decreased likelihood of adopting BLW (18.1% vs. 25.7%; *p*-value = 0.115). The possible causal pathway—that parental eating may only influence feeding difficulties in a direct way (solid line), independently from possible mediators—was confirmed in a conditional analysis that also includes the mediators. Parental eating still revealed an effect on feeding difficulties (both parents omnivorous: 0.46 (0.30–0.70), *p* < 0.0001; BF duration ≥9 months: 0.78 (0.60–1.02), *p* = 0.065; BLW: 0.72 (0.52–1.00), *p*-value = 0.052).

### 3.4. Feeding Difficulty Score and Weight and Pressure Categories

LR, IR, and HR children were compared in terms of anthropometric variables and blood pressure. We observed that the BMI percentile was significantly lower in HR children (48.5 ± 30.3) compared to both IR (51.1 ± 32.6) and LR children (56.1 ± 32.6; *p* = 0.04). When examining the prevalence of atypical behavior risks across the different BMI categories, we found that underweight children tended to have a higher probability of being classified as IR or HR rather than LR, with proportions of 15.4%, 12.2%, and 8.5%, respectively. However, no significant differences were found across the weight categories. When examining the prevalence of atypical behavior risks across the different BP categories, i.e., normal BP and hypertension, we did not assess any difference. The relationship between the hypertension status and all the parameters considered in Table 2 and Table 4 was non-significant.

## 4. Discussion

The current study explored parent-reported feeding difficulties and their association with socio-demographic factors and early feeding history in a population of Italian primary school children. More than 60% of the children were at intermediate to high risk. Although not specifically on food neophobia, our results agreed with those from Italian studies showing high levels of food neophobia in middle childhood [7,8]. Notably, our findings highlighted a high level of clustering of feeding difficulties, refusal, selective eating, and monotony. Nearly two thirds of parents reported that their child refused certain foods, whilst one third reported that their child struggled with vegetables. These patterns suggested a risk of nutritional imbalances and inadequacies in this population. Feeding difficulties, characterized by avoidance/rejection of certain food groups or limited food selection, can lead to low dietary variety, failure to meet dietary recommendations, and potential emergence of later eating disorders. Our results aligned with previous research in this area. Summarizing the existing evidence, several authors found that picky eaters consumed fewer fruits and vegetables, whole grains, meat, and fish than non-picky eaters [15,16]. In 9–12-year-old children from five European countries (Finland, Italy, Spain, Sweden, and the UK), food neophobia was negatively associated with liking wholegrain biscuits and the consumption of fruits, vegetables, and wholegrain products [6]. The Irish ROLO longitudinal study showed that ‘Food Fussiness’ was inversely linked to the Healthy Eating Index in children at 5 and 9–11 years of age [17]. In Italian children aged 3–11 years, food neophobia predicted low adherence to Mediterranean diet by reducing the consumption of fruit, vegetables, legumes, and nuts and by increasing that of sweets and fast food [7]. A recent meta-analysis showed that picky eating was positively associated with being underweight [16]. Our data reflected these trends: underweight children showed a tendency towards a higher probability of being at IR or HR rather than LR; HR children had significantly lower BMI percentiles compared to both the IR and LR groups.

Socio-demographic analyses revealed the influence of parental eating on child eating in our population. Specifically, we found that parental eating directly influenced child feeding difficulties, independently from possible mediators. Our findings suggest that parents served as primary role models [18]. Participants with at least one non-omnivorous parent showed higher feeding difficulty scores, and a higher probability of belonging to the HR group. By contrast, LR participants more often had two omnivorous parents. Interestingly, parental eating influenced feeding difficulties only in a direct way, independently from possible mediators. These patterns aligned with evidence showing that children tend to adopt their parents’ dietary behaviors and preferences, and parental modeling is consistently associated with children’s healthy and unhealthy food consumption [19,20]. Parents influence children’s eating habits directly, by shaping the home food environment, and indirectly, through their own dietary patterns. Children tend to adopt healthier eating behaviors when their parents model them. For example, a New Zealand survey of parent–child dyads found that a one-unit increase in parental diet quality was associated with a 0.03 SD reduction in children’s “Snacks” scores [21]. A meta-analysis of 37 studies identified food availability and parental modeling as the strongest predictors of children’s food consumption [22]. In contrast, controlling feeding practices, including restriction, pressure to eat, or using food as a reward, have been associated with less healthy growth patterns, food preferences, and dietary habits [20,23,24], whereas supportive feeding practices appear to promote healthier eating [20,23]. However, Pickard and colleagues [24] reported that lower encouragement of dietary balance and variety among UK parents of children aged 3–6 years was linked to a higher likelihood of typical eating behaviors. This implies that promoting dietary variety alone may be insufficient to ensure the development of typical or optimal eating patterns; instead, feeding approaches may need to be tailored to children’s specific eating profiles. To our knowledge, the present research is the first study to link children’s feeding difficulties to omnivorous parents or non-omnivorous parents. For example, the Avon Longitudinal Study of Parents and Children, investigating the development of picky eating in childhood and the effects on diet adequacy and growth [25] did not stratify children by parental dietary patterns. We observed higher feeding difficulty scores in children without siblings, in line with Di Nucci et al. [7], who reported a lower proportion of only children among neophilic compared with neophobic children. Although siblings are thought to influence children’s eating behaviors through modeling and shared parental feeding practices [26,27], sibling presence was not significantly associated with feeding difficulties in our sample. This contrasts with evidence from the Portuguese Generation XXI cohort [28] and from the US Early Childhood Longitudinal Study—Kindergarten [29], which reported more favorable dietary behaviors in children living with siblings. We also found that children who were breastfed for less than 9 months showed higher feeding difficulty scores, more monotonous diets, and greater difficulties with vegetables than those who were breastfed for longer. Similar patterns were observed among children who were spoon-fed. Although the associations between longer breastfeeding duration (at least 9 months), BLW, and later feeding difficulties did not reach statistical significance, the effects were borderline. These findings are consistent with the broader literature, which reports a general lack of consistent evidence for associations between early feeding practices and parent-reported feeding difficulties beyond infancy [1,30]. Overall, our results suggest that infant feeding practices may represent one of several factors contributing to feeding difficulties in middle childhood, potentially through early exposure to a wider range of tastes and textures. Prolonged breastfeeding may expose infants to variability in breast milk flavor, while the introduction of diverse complementary foods may enhance familiarity with a range of tastes and textures, thereby supporting later food acceptance [31]. Evidence from an umbrella review further indicates that repeated early exposure to vegetables increases children’s liking and intake [32]. Together, these findings support recommendations to continue breastfeeding beyond 6 months while introducing a variety of complementary foods that are in line with children’s self-regulatory capacities [33].

They also highlight that a child’s diet depends on whether the family follows healthy eating.

In the light of our results, health professionals should encourage daily consumption of fruit and vegetable and provide tailored guidance to support nutritionally balanced diets and address child feeding difficulties. Interventions such as taste, sensory, and culinary education, cooking activities, and gardening have been shown to reduce food neophobia by increasing exposure, familiarity, curiosity, and positive food-related experiences [34]. Meta-analyses examining parent-targeted, home-based interventions further indicate that repeated taste exposure effectively increases short-term vegetable intake, whereas nutritional education is more strongly associated with improvements in fruit consumption [35]. Overall, managing feeding difficulties requires a multifaceted approach: offering small and regular meals; repeatedly presenting foods without pressure, complaints, or food-based rewards; keeping foods accessible; and introducing new items alongside familiar ones, including through food chaining [36]. Involving children in meal preparation is another key strategy for increasing the willingness to consume fruits and vegetables. Integrating family-centered, responsive feeding approaches into routine pediatric care, alongside supportive public policies that enhance parental education and access to healthy foods, particularly in socioeconomically vulnerable contexts, is essential for promoting positive feeding environments [37].

### Limits and Strengths

This research presents some limitations. The cross-sectional design does not allow for establishing causal relationships. Voluntary participation might have introduced a selection bias, as participants might not fully represent the broader population (for example, in terms of interest or awareness). The reliance on parent-reported questionnaires and parental recall of early-life feeding practices might have introduced reporting biases, as responses could have been influenced by the misinterpretation of questions, subjective judgment, or inaccurate memory. Assessments of child’s eating difficulties were based on parental perception, which may not accurately reflect actual behavior. The study did not collect detailed socioeconomic information, such as parental income, employment status, type of job, and neighborhood of residence, which may affect the generalization of results. The newly developed questionnaire has not undergone psychometric validation, which represents a further limitation. Lastly, we did not assess parents’ own eating habits or dietary history, which could have provided important insights for characterizing the heterogeneous group of non-omnivorous families. However, to design such a tool, we paid particular attention to the vocabulary and the list of questions to avoid a lengthy and unclear questionnaire, which could compromise the completion rates. The scoring questions in the questionnaire demonstrated high internal consistency, indicating adequate reliability or uniformity in results. To date, there is limited information about overall feeding difficulties in middle childhood in Italy, with the existing studies dealing with food neophobia, which represents only a portion of the broader spectrum of feeding challenges. Likewise, globally, there is no consistent evidence yet that early feeding practices contribute to parent-reported feeding difficulties in children older than 1 year. Therefore, our study, which investigated a wider range of eating difficulties, may contribute to addressing such a gap in knowledge and providing pediatricians with suggestions that are useful to handle the above-mentioned challenge. Another strength of this work is the sample size, ensuring sufficient statistical power to detect meaningful differences or associations between variables and the precision of estimates. In addition, participants were recruited from several Italian regions, resulting in an overall geographical distribution that was broadly consistent with national data on the distribution of children aged 5–11 years in 2024 (Northern Italy: 46,4%; Central Italy: 19.4%; and Southern Italy: 34.3), thereby supporting a reasonable level of representativeness of the sample [38]. Furthermore, a broad spectrum of factors was taken into account, thus providing a holistic view of the issue.

## 5. Conclusions

Childhood eating behavior can potentially affect health in the short and long term. Delays in identifying feeding difficulties among children can increase the risk of malnutrition, impaired growth and development, and eating disorders. This pilot study confirms that feeding difficulties are widespread in Italian children aged 5–11 years old. Having two omnivorous parents appeared to be defensive towards feeding difficulties and the risk of atypical feeding behaviors, while longer breastfeeding duration (≥9 months) and baby-led weaning showed borderline defensive associations. These findings indicate the role of parental eating in shaping children’s eating behaviors, highlighting the importance of varied dietary patterns across life to prevent lifelong atypical eating behaviors and nutrition-related consequences. Moreover, they support the importance of early, family-centered nutritional education. Family pediatricians can play a relevant role in monitoring and counseling parents/caregivers to promote healthy, pressure-free eating environments and experiential food learning. Further research is needed to validate the newly developed questionnaire and to conduct longitudinal studies including households’ socioeconomic characteristics in order to validate and extend the findings, as well as to better understand feeding difficulties in childhood. This could help guide implement effective targeted strategies to avert the onset of childhood food difficulties.

## Figures and Tables

**Table 1:** General characteristics, feeding history, anthropometry, and blood pressure (BP) of the participants (*n* = 742).

Participants	Variables	Mean (±sd)	*N* (%)
**Child**	**Age**	7.5 (±1.6)	
	**Born preterm (<37 weeks)**		69 (9.3)
	**Only child**		185 (24.9)
	**First-born child**		425 (57.3)
	**Duration of breastfeeding (months)**		
	0–<1		138 (18.6)
	1–3		96 (12.9)
	3–6		88 (11.9)
	6–9		92 (12.4)
	9–12		104 (14.0)
	>12		224 (30.2)
	**Complementary feeding method**		
	Traditional baby weaning or spoon-fed weaning		598 (80.6)
	Baby-led weaning or auto-weaning		140 (18.9)
	Do not remember		4 (0.5)
	**Weight (kg)**	26.4 (±7.6)	
	**Height (cm)**	125.3 (±10.9)	
	**Body mass index (BMI)**	16.5 (±2.8)	
	**BMI percentile**	52.4 (±31.1)	
	**Nutritional status ^†^** **:**		
	Underweight		91 (12.3)
	Normal weight		484 (65.2)
	Overweight		107 (14.4)
	Obese		60 (8.1)
	**Systolic BP ^#^ (mmHg)**	96.1 (±10.7)	
	**Systolic BP ^#^ percentile**	48.0 (±29.6)	
	**Diastolic BP ^#^ (mmHg)**	60.2 (±9.7)	
	**Diastolic BP ^#^ percentile**	56.3 (±26.0)	
	**BP ^#^ categories °**		
	Normal BP		652 (87.9)
	Hypertension		86 (11.6)
**Mother**	**Italian nationality**		666 (89.8)
	**Graduate**		313 (42.2)
	**Omnivore**		694 (93.5)
**Father**	**Italian nationality**		689 (92.9)
	**Graduate**		187 (25.2)
	**Omnivore**		699 (94.2)

^†^ According to the International Obesity Task Force (underweight <10th percentile; overweight > 85th percentile; obese > 95th percentile) [10]. ^#^ Four missed. ° According to the American Academy of Pediatrics [11].

**Table 2:** Prevalence [*n* (%)] of eating behaviors related to difficulties, refusal, selectivity, and monotony among 742 children participating in the study, with the first 10 questions (in bold) being taken into account to calculate the score.

Eating Behavior	*N* (%)		
	Yes	No	Do Not Reply
Does your child eat almost everything?	509 (68.6)	**228 (30.7)**	5 (0.7)
2. Does your child only eat a few foods?	**188 (25.3)**	537 (72.4)	17 (2.3)
3. Does your child always eat the same foods?	**246 (33.1)**	488 (65.8)	8 (1.1)
4. Does your child absolutely refuse certain foods?	**458 (61.7)**	274 (36.9)	10 (1.4)
5. Does your child have a hard time eating vegetables or refuse them?	**276 (37.2)**	452 (60.9)	14 (1.9)
6. Does your child always like the same methods of preparing food (e.g., plain pasta)?	**237 (31.9)**	495 (66.7)	10 (1.4)
7. Does your child show strong resistance or refuse to try new foods?	**231 (31.1)**	499 (67.3)	12 (1.6)
8. Does your child have any likes or dislikes that force you to prepare meals that are different from those of the rest of the family?	**238 (32.1)**	493 (66.4)	11 (1.5)
9. Does your child only eat unwanted foods if hidden in other foods?	**198 (26.7)**	504 (67.9)	40 (5.4)
10. Does your child take more than 30 min to finish a meal?	**183 (24.7)**	549 (74.0)	10 (1.3)
11. Does your child eat everything in the school canteen? ^#^	466 (77.7)	115 (19.2)	19 (3.2)
12. Does your child behave differently depending on which family member offers them food?	124 (16.7)	613 (82.6)	5 (0.7)
13. Do you sometimes buy food for your child for reasons other than simple food choices (e.g., gadgets, games, advertising campaigns)?	182 (24.5)	547 (73.7)	13 (1.8)
14. Does your child have difficulty eating foods that have a particular texture, such as those that require chewing?	53 (7.1)	681 (91.8)	8 (1.1)
15. Does your child only eat when distracted by games, TV, videos, etc.?	74 (10.0)	668 (90.0)	0 (0.0)

^#^ The prevalence was calculated among 600 children attending the school canteen.

**Table 3:** Differences in the feeding difficulty score [mean (±sd)] based on children’s general characteristics and feeding history.

Children’s Characteristics (*n*)	Feeding Difficulty Score	*p*-Value
Age: <7.5 years (383) vs. ≥7.5 years (359)	3.5 (±3.1) vs. 3.2 (±2.9)	0.3952
Born at term (673) vs. Born pre-term (69)	3.4 (±3.0) vs. 3.1 (±3.1)	0.4234
Only child (185) vs. Having siblings (557)	3.7 (±2.9) vs. 3.2 (±3.0)	0.0397
First-born child (425) vs. Not first-born child (317)	3.5 (±3.0) vs. 3.1 (±3.0)	0.0741
Breastfeeding duration: ≥9 months (328) vs. < 9 months (414)	3.0 (±2.9) vs. 3.6 (±3.1)	0.0221
Traditional spoon-fed weaning (598) vs. Baby-led weaning (140)	3.4 (±3.0) vs. 2.9 (±2.9)	0.0333
Italian mother (666) vs. Not Italian mother (76)	3.3 (±3.0) vs. 3.7 (±3.0)	0.1808
Graduate mother (313) vs. Not graduate mother (429)	3.2 (±3.0) vs. 3.4 (±3.0)	0.1386
Omnivorous mother (694) vs. Non-omnivorous mother (48)	3.3 (±2.9) vs. 4.6 (±3.3)	0.0068
Italian father (689) vs. Not Italian father (53)	3.3 (±3.0) vs. 3.6 (±3.0)	0.4196
Graduate father (187) vs. Not graduate father (555)	3.4 (±3.1) vs. 3.3 (±2.9)	0.9744
Omnivorous father (699) vs. Non-omnivorous father (43)	3.2 (±2.9) vs. 4.8 (±3.3)	0.0020
Both parents omnivorous (668) vs. At least one non-omnivorous parent (74)	3.2 (±2.9) vs. 4.6 (±3.2)	0.0006

All *p*-values from two-sample Wilcoxon rank-sum (Mann–Whitney test).

**Table 4:** Frequencies [*n* (%)] and differences in children’s general characteristics and feeding history according to the type of atypical behavior risk.

Children’s Characteristics (*n*)	Children at Risk of Atypical Behavior			
	HR (Score ≥ 7)	IR (Score 2–6)	LR (Score ≤ 1)	*p* Value
Age: <7.5 years (383) vs. ≥7.5 years (359)	83 (21.7) vs. 64 (17.8)	163 (42.6) vs. 135 (45.1)	137 (35.8) vs. 133 (37.0)	0.418
Born pre-term (69) vs. Born at term (673)	12 (17.4) vs. 135 (20.1)	26 (37.7) vs. 299 (44.4)	31 (44.9) vs. 239 (35.5)	0.301
Only child (185) vs. Having siblings (557)	37 (20.0) vs. 110 (19.7)	93 (50.3) vs. 232 (41.6)	55 (29.7) vs. 215 (38.6)	0.068
First-born child (425) vs. Not first-born child (317)	86 (20.2) vs. 61 (19.2)	195 (45.9) vs. 130 (41.0)	144 (33.9) vs. 126 (39.7)	0.248
Breastfeeding duration: <9 months (414) vs. ≥9 months (328)	95 (22.9) vs. 52 (15.8)	174 (42.0) vs. 151 (46.0)	145 (35.0) vs. 125 (38.1)	0.055
Traditional spoon-fed weaning (602) vs. Baby-led weaning (140)	124 (20.6) vs. 23 (16.4)	271 (45.0) vs. 54 (38.6)	207 (34.4) vs. 63 (45.0)	0.061
Italian mother (666) vs. Not Italian mother (76)	128 (19.2) vs. 19 (25.0)	293 (44.0) vs. 32 (42.11)	245 (36.8) vs. 25 (32.9)	0.475
Graduate mother (313) vs. Not graduate mother (429)	63 (20.1) vs. 84 (19.6)	130 (41.5) vs. 195 (45.4)	120 (38.3) vs. 150 (35.0)	0.541
Omnivorous mother (694) vs. Non-omnivorous mother (48)	129 (18.6) vs. 18 (37.5)	306 (44.1) vs. 19 (39.6)	259 (37.3) vs. 11 (22.9)	0.004
Italian father (689) vs. Not Italian father (53)	135 (19.6) vs. 12 (22.6)	298 (43.2) vs. 27 (50.9)	256 (37.2) vs. 14 (26.4)	0.293
Graduate father (197) vs. Not graduate father (555)	43 (23.0) vs. 104 (18.7)	75 (40.1) vs. 250 (45.0)	69 (37) vs. 201 (36.9)	0.354
Omnivorous father (699) vs. Non-omnivorous father (43)	133 (19.0) vs. 14 (32.6)	303 (43.3) vs. 22 (51.2)	263 (37.6) vs. 7 (16.3)	0.009
At least one non-omnivorous parent (74) vs. Both parents omnivorous (668)	25 (33.8) vs. 122 (18.3)	33 (44.6) vs. 292 (43.7)	16 (21.6) vs. 254 (38.0)	0.001

All *p*-values from Pearson’s chi-squared test. HR: high risk; LR: low risk; and IR: intermediate risk.

**Table 5:** Independent variables included in the ordinal logistic regression model and their logarithm odds ratios (OR), 95% confidence intervals (CI), and *p*-values. The dependent variable was the feeding difficulty score.

Children at Risk of Atypical Behavior	OR (95% CI)	*p* Value
Only child	1.15 (0.82–1.60)	0.423
First-born child	1.20 (0.89–1.61)	0.237
Breastfeeding duration ≥9 months	0.79 (0.61–1.02)	0.075
Baby-led weaning	0.72 (0.52–1.00)	0.053
Both parents omnivorous	0.46 (0.30–0.71)	<0.0001

## Data Availability

All the raw data presented in this study can be provided upon request by the corresponding author.

## Abbreviations

The following abbreviations are used in this manuscript:

SICuPP	Società Italiana delle Cure Primarie Pediatriche (Italian Society of Pediatric Primary Care)
BMI	Body mass index
IOTF	International Obesity Task Force
BP	Blood pressure
SBP	Systolic pressure
DBP	Diastolic pressure
CEBQ	Child Eating Behavior Questionnaire
LR	Low risk
IR	Intermediate risk
HR	High risk
BF	Breastfeeding
BLW	Baby-led weaning
